# Comparison of cardiovascular co-morbidities and CPAP use in patients with positional and non-positional mild obstructive sleep apnea

**DOI:** 10.1186/1471-2466-14-153

**Published:** 2014-09-26

**Authors:** Yi-Chih Huang, Chun-Yao Lin, Chou-Chin Lan, Yao-Kuang Wu, Chor-Shen Lim, Chun-Yao Huang, Hsuan-Li Huang, Kuan-Hung Yeh, Yu-Chih Liu, Mei-Chen Yang

**Affiliations:** Division of Pulmonary Medicine, Department of Internal Medicine, Taipei Tzuchi Hospital, the Buddhist Tzuchi Medical Foundation, Xindian Dist, New Taipei City, Taiwan; School of Medicine, Tzu-Chi University, Hualien, Taiwan; Divisions of Pulmonary, Critical Care and Sleep Medicine, Chang Gung Memorial Hospital at Keelung, Keelung City, Taiwan; Division of Cardiology, Department of Internal Medicine, Taipei Tzuchi Hospital, the Buddhist Tzuchi Medical Foundation, Xindian Dist, New Taipei City, Taiwan

**Keywords:** Obstructive sleep apnea, Positional sleep apnea, Cardiovascular co-morbidities, Hypertension, Continuous positive airway pressure

## Abstract

**Background:**

This retrospective cohort study aimed to determine if there are differences in cardiovascular co-morbidities, blood pressure (BP) and continuous positive airway pressure (CPAP) use between patients with positional-dependent and nonpositional-dependent obstructive sleep apnea (OSA).

**Methods:**

Patients who were referred for overnight polysomnography for suspected OSA between 2007 and 2011 were screened. A total of 371 patients with OSA were included for analysis and divided into six groups according to positional-dependency and severity of OSA: positional mild (n = 52), positional moderate (n = 29), positional severe (n = 24), non-positional mild (n = 18), non-positional moderate (n = 70) and non-positional severe group (n = 178). The six groups were compared for anthropometric and polysomnographic variables, presence of cardiovascular co-morbidities, morning and evening BP and the changes between evening and morning BP, and CPAP device usage patterns.

**Results:**

Demographic and anthropometric variables showed non-positional severe OSA had poor sleep quality and higher morning blood pressures. Positional mild OSA had the lowest cardiovascular co-morbidities. Overall CPAP acceptance was 45.6%. Mild OSA patients had the lowest CPAP acceptance rate (10%), followed by moderate group (37.37%) and severe group (61.88%, *P* < 0.001). However, the significant difference in CPAP acceptance across OSA severity disappeared when the data was stratified by positional dependency.

**Conclusions:**

This study found that positional mild OSA had less cardiovascular co-morbidities compared with subjects with positional severe OSA. Independent of posture, CPAP acceptance in patients with mild OSA was low, but CPAP compliance was similar in CPAP acceptors regardless of posture dependency of OSA. Since there are increasing evidences of greater cardiovascular risk for untreated mild OSA, improving CPAP acceptance among mild OSA patients may be clinically important regardless of posture dependency.

## Background

Severe obstructive sleep apnea (OSA) is associated with an increased risk of cardiovascular (CV) disease, including coronary artery disease, heart failure, and stroke [1]. Although often overlooked, mild OSA has been associated with a higher prevalence of CV disease and significant CV co-morbidities, including hypertension and carotid artery atherosclerosis [2–5]; although, other studies have not found an association of OSA with hypertension and CV diseases [6]. As the severity of mild OSA tends to worse over time, active and effective treatment for mild OSA may be required [7].

The vast majority of patients with mild OSA exhibit position-dependent apnea, in which the presence and severity of symptoms are related to body position, and the associated gravitational changes, during sleep [8, 9]. Specifically, gravity pulls the jaw and the tongue downwards while in the supine position, partially or fully obstructing the airway. Positional OSA is generally indicated by a total apnea-hypopnea index (AHI) ≥ 5, with a > 50% reduction in the AHI between the supine and lateral positions, and an AHI that normalizes (AHI < 5) in the lateral position [9].

Various treatment modalities are available for mild OSA. Positional therapy, aimed at maintaining a non-supine sleep position, is often used as first-line treatment for patients with positional mild OSA, but has only moderate efficacy and poor compliance [10]. Continuous positive airway pressure (CPAP) treatment significantly reduces the AHI and improves sleep efficiency in patients with OSA [11]; however both patients with mild OSA and their clinicians are significantly less inclined to accept CPAP treatment [11]. Mandibular advancement devices, while generally preferred by patients over CPAP, are not as effective in reducing sleep apnea symptoms as CPAP [11]. In addition to its proven efficacy for improving apnea symptoms, effective CPAP treatment is associated with significantly decreased risks for cardiovascular disorders, including reduced arterial stiffness and decreased blood pressure (BP) in patients with OSA [12–14].

Few studies have examined BP and CPAP use in patients with positional-dependent mild OSA. Therefore, the primary aim of this study was to determine if there are differences in BP (including morning BP, evening BP, and the different in BP between evening and morning) between patients who have positional and non-positional mild OSA. We also evaluated patients with mild, moderate, and severe OSA. CPAP usage patterns in these patients were also evaluated.

## Methods

### Study subjects

A total of 874 consecutive adult patients (aged ≥ 18 years) were screened in this retrospective cohort study. All were referred to the Chest and Sleep Clinic of Taipei Tzuchi Hospital, the Buddhist Tzuchi Medical Foundation (New Taipei City, Taiwan) for overnight polysomnography (PSG) for suspected OSA between January 2007 and December 2011. None of the subjects were previously diagnosed with OSA. Patients who underwent split-night sleep studies were excluded (n = 46) due to the inherent difficulty in separating the diagnostic and therapeutic portions of the study for postural effects [15]. Patients with PSG results that did not include at least 15 minutes of data obtained in both supine and non-supine positions were also excluded (n = 352) [15]. This time period was chosen with reference to a previous study [15] and did not have to be consecutive. Patients with PSG results indicating non-apnea (AHI < 5/h) were excluded (n = 103). We also excluded two patients whose PSG data was missing. The remaining 371 patients with OSA (AHI ≥ 5) were included in the final analyses. These 371 patients were divided into six groups by positional-dependency of OSA and the severity of OSA. Positional OSA was defined as a > 50% reduction in the AHI between the supine and lateral positions and an AHI that normalized (AHI < 5) in a non-supine sleep position. This study was approved by the Institutional Review Board of the Taipei Tzuchi Hospital, the Buddhist Tzuchi Medical Foundation. The informed patient consent was waived since this was retrospective study.

### Measurements

#### Anthropometric measurements and demographic data

Medical history and anthropometric data were recorded prior to PSG study, and included body weight, height, body mass index (BMI), neck, waist, and hip circumference, waist-to-hip ratio, smoking status, hypertension, anti-hypertensive agents usage, and presence of CV diseases, defined as previous diagnosis of coronary artery disease (CAD) or a history of cerebrovascular accident (CVA).

#### Sleep parameters

Excessive daytime sleepiness was evaluated using the Chinese version of the Epworth Sleepiness Scale (ESS) before overnight PSG study [16]. An attended, standard overnight PSG study was performed by trained sleep technicians at the sleep center. During PSG, electroencephalography (EEG), electrooculography, chin and bilateral anterior tibialis surface electromyography, electrocardiography, airflow through the nose and mouth (registered by thermistor), thoracoabdominal movements (registered by respiratory inductive plethysmography), position (by a sensor on the respiratory inductive plethysmography), snoring, and oxygen saturation (by pulse oximetry) were recorded. The PSG study lasted for at least 6 hours. PSG data were analyzed by manual scoring for every 30-second epoch by trained sleep technicians and were reviewed by sleep specialists.

Sleep stage was scored by trained sleep technicians using the standard criteria of Rechtschaffen and Kales [17]. An apnea event was defined as an 80-100% reduction in airflow lasting for at least 10 seconds. A hypopnea event was defined as a reduction in airflow of at least 50% for at least 10 seconds or at least a 30% reduction in airflow for at least 10 seconds as compared with baseline and associated with at least 3% oxygen desaturation or with an EEG arousal. AHI was calculated from the total number of apnea and hypopnea events per hour of sleep. The desaturation index (DI) was defined as > 3% oxygen desaturation per hour of sleep. The arousal index (AI) was defined as arousal episodes per hour of sleep [18]. Sleep efficiency was defined as the fraction of total sleep time to total recording time. Sleep latency was defined as the time from lights off to the first identifiable sleep stage. Rapid eye movement (REM) latency was defined as the time from the first identifiable sleep stage to the first REM sleep.

#### Blood pressure

BP was measured with the patient in the supine position by trained technicians using an automated sphygmomanometer (Welch Allyn Vital Signs Monitor 300 Series) with an optimal cuff. The automated sphygmomanometer was regularly calibrated at least every year by the technician from the manufacturer who calibrated and/or validated the instrument against manual measurement performed with a standard mercury sphygmomanometer. Evening BP was measured after 15 minutes of rest and before sleep onset. Morning BP was measured immediately upon awakening with the patient attached to all PSG equipments. Two consecutive determinations were made on each occasion, separated by 5 minutes, and the results were averaged for both the evening and morning BP readings. Mean arterial blood pressure (MABP) was calculated using the systolic BP (SBP) and diastolic BP (DBP): MABP = 1/3 SBP + 2/3 DBP. Evening-to-morning BP difference was determined by: morning BP – evening BP.

#### CPAP acceptance and compliance

All patients were offered treatment with CPAP. CPAP acceptance refers to the proportions of patients who meet the selection criteria for CPAP treatment and were willing to try CPAP and actually to use it at home [19, 20]. CPAP compliance refers to the proportions of patients using CPAP and delivering a pre-set level over a given time period [19, 20] Patients were routinely followed up every 3 months at the outpatient clinic. According to the policy of our sleep center, we routinely collected objective CPAP usage data (recorded by the device software) at each visit. The CPAP usage included percentage of days used, percentage of nights during which CPAP was used for ≥ 4 hours, and the overall mean hours used per night.

High CPAP compliance was defined as ≥ 4 hours of CPAP per night for ≥ 70% of the nights [21]. Patients who did not meet these levels of CPAP usage were defined as having low CPAP compliance.

#### Statistical analysis

Results for continuous variables are presented as mean ± standard deviation, whereas results for categorical variables are presented as number (percentage). A Kolmogorov-Smirnov test was used to test for normality. Baseline characteristics between the positional OSA and non-positional OSA groups were compared by one-way analysis of variance (ANOVA) (continuous variables) or chi-square/Fisher’s exact test (categorical variables). When significant results were revealed by ANOVA, post-hoc tests with Bonferroni correction or Dunnett’s test were then carried out. Daytime sleepiness measurements, overnight polysomnography and blood pressure results among groups adjusting for baseline differences were estimated by linear regression models. All statistical assessments were evaluated at a two-sided alpha level of 0.05 using SAS software, version 9.2 (SAS Institute, Inc., Cary, NC, USA).

## Results

### Baseline characteristics and anthropometric measurements

**Figure 1:**
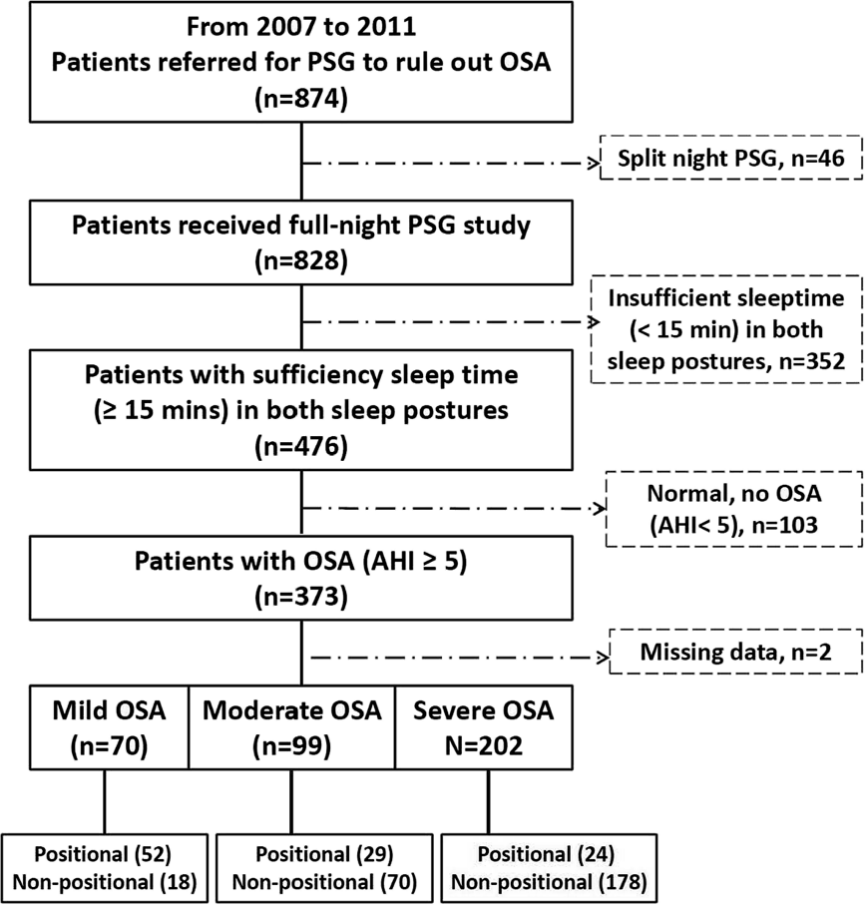
**Flowchart of patient enrollment.** PSG: polysomnography; OSA: obstructive sleep apnea; AHI: apnea-hypopnea index.

The screening results are summarized in Figure 1. A total of 874 adult patients met the inclusion criteria for screening. Of these, 46 were excluded for undergoing split-night studies, 352 for not having sufficient data in both supine and non-supine sleep positions, 103 for having normal AHI, and 2 for missing PSG data.

The 371 patients who were included in the analysis were divided into six groups: non-positional mild OSA (group 1, n = 18), non-positional moderate OSA (group 2, n = 70), non-positional severe OSA (group 3, n = 178), positional mild OSA (group 4, n = 52), positional moderate OSA (group 5, n = 29), and positional severe OSA (group 6, n = 24).

The baseline characteristics of these six groups are summarized in Table 1. Except for age, CAD and CVA, differences in gender, neck circumference, waist circumference and hip circumference, ratio of waist to hip, BMI, smoking history, CV co-morbidities, hypertension and anti-hypertensive drug use among groups were found. There were more males than females in the study with the highest proportion of males having severe OSA in both the non-positional and positional groups. Subjects with severe OSA in the non-positional subgroup had greater waist and hip circumference, higher waist-to-hip ratio, and BMI than the subjects with severe OSA than in the positional group. The neck circumference, waist circumference, and hip circumference in subjects with severe OSA (Group 3) in the non-position cohort were larger compared with those with mild (Group 1) or moderate (Group 2) OSA. There was no difference in these same variables across disease severity in the positional cohort. A similar percentage of patients had hypertension between the non-positional and positional cohort of subjects.

**Table 1:** **Baseline characteristics of patients with non-positional and positional obstructive sleep apnea**

	Non-positional			Positional			P-value
	G1: Mild	G2: Moderate	G3: Severe	G4: Mild	G5: Moderate	G6: Severe	
	(n = 18)	(n = 70)	(n = 178)	(n = 52)	(n = 29)	(n = 24)	
Age, years	61.39 ± 15.27	60.9 ± 13.06	56.39 ± 13.54	56.55 ± 13.2	54.76 ± 10.5	55.46 ± 13.95	0.999
Gender							0.011*
Female	6(33.33%)	25(35.71%)	34(19.1%)^b^	18(34.62%)	6(20.69%)	2(8.33%)^a^	
Male	12(66.67%)	45(64.29%)	144(80.9%)	34(65.38%)	23(79.31%)	22(91.67%)	
Neck circumference, cm	37.83 ± 3.63	38.22 ± 3.05	40.55 ± 3.7^a,b^	37.28 ± 3.05	38.66 ± 2.79	38.58 ± 2.62	<0.001*
Waist circumference, cm	92.64 ± 10.66	97.24 ± 9.08	103.21 ± 11.67^a,b^	91.99 ± 10.59	95.71 ± 8.34	92.64 ± 5.51^c^	<0.001*
Hip circumference, cm	99.36 ± 9.95	103.43 ± 9.31	107.4 ± 10.55^a,b^	100.73 ± 7.5	102.71 ± 6.69	100.02 ± 4.99^c^	<0.001*
Waist-to-hip ratio, %	0.93 ± 0.06	0.94 ± 0.05	0.96 ± 0.05	0.91 ± 0.07	0.93 ± 0.05	0.92 ± 0.05^c^	<0.001*
BMI, kg/m^2^	26.27 ± 4.78	27.58 ± 4.76	29.98 ± 5.19	26.29 ± 3.41	27.74 ± 2.96	26.15 ± 2.78^c^	<0.001*
Smoking history							0.017*
Past-smoker	4(22.22%)	14(20%)	42(23.6%)	9(17.31%)	6(20.69%)	11(45.83%)^a^	
Never smoked	8(44.44%)	46(65.71%)	83(46.63%)	34(65.38%)	17(58.62%)	11(45.83%)	
Current smoker	6(33.33%)	10(14.29%)	53(29.78%)	9(17.31%)	6(20.69%)	2(8.33%)	
CV co-morbidities							
Any CV co-morbidity	11(61.11%)	39(55.71%)	113(63.48%)	19(36.54%)	15(51.72%)	17(70.83%)^a^	0.018*
HTN	11(61.11%)	38(54.29%)	111(62.36%)	18(34.62%)	15(51.72%)	16(66.67%)^a^	0.015*
Anti-HTN drugs^†^	11(100%)	37(97.37%)	89(80.18%)^b^	18(100%)	13(86.67%)	14(87.5%)	0.021*
Dosage of anti-HTN drugs (number of tablets /day)^†^	2.23 ± 1.40	2.21 ± 1.15	1.98 ± 1.14	1.74 ± 1.24	1.35 ± 0.52	1.77 ± 0.92	0.192
CAD	0(0%)	3(4.29%)	13(7.3%)	0(0%)	1(3.45%)	2(8.33%)	0.394
CVA	2(11.11%)	15(21.43%)	32(17.98%)	4(7.69%)	6(20.69%)	5(20.83%)	0.307

BMI: body mass index; CV: cardiovascular; HTN: hypertension; CAD: coronary artery disease; CVA: cerebrovascular accident.

Results are mean ± standard deviation for continuous variables and number (percentage) for categorical variables.

Intergroup comparisons were made by *analysis of variance* followed by Bonferroni correction or Dunnett’s test for post-hoc test (continuous variables) or Chi-square / Fisher’s exact tests (categorical variables).

*Indicates a significant between group difference, *P* < 0.05.

^†^Only HTN patients were analyzed.

^a^Indicates significant difference from the mild subgroup within the nonpositional (or positional) group, *p* < 0.05.

^b^Indicates significant difference from the moderate subgroup within the nonpositional (or positional) group, *p* < 0.05.

^c^Indicates significant difference between the corresponding subgroups of nonpositional and positional groups, *p* < 0.05.

### Sleep parameters

Table 2 summarizes the daytime sleepiness measurements and the overnight PSG results. ESS scores were not significantly different across groups. After adjusting for gender, waist-to-hip ratio, BMI, smoking and cardiovascular co-morbidity, significant associations were found for AHI, supine AHI, lateral AHI, DI, AI, S1, S2, REM, SaO_2_ mean, and SaO_2_ minimal (all *P* < 0.001). Patients with non-positional severe OSA had higher AHI (55.98 ± 1.01 episodes/hour), supine AHI (66.11 ± 1.16 episodes/hours), lateral AHI (39.29 ± 1.35 episodes/hours) and lower SaO_2_ mean (90.65% ± 0.24%) than those with positional OSA. Within the non-positional group, group 3 had the largest values of the number of AHI, supine AHI, lateral AHI, DI, AI, and S1, followed by group 2 than group 1 who were similar in respect to the number of lateral AHI, DI, AI, and S1. S2, SaO_2_ mean, and SaO_2_ minimal in group 3 were smaller than those in the other two subgroups. In the positional group, the frequency of AHI and supine AHI were different among three subgroups in order of G6 > G5 > G4. The number of DI and AI in both group 4 and group 5 were smaller than those in the group 6. The SaO_2_ minimal was significantly different only between the group 4 and group 6.

**Table 2:** **Comparison of daytime sleepiness measurements, overnight polysomnography and blood pressure results for patients with non-positional and positional obstructive sleep apnea**

	Non-positional			Positional			Adjusted
	G1: Mild	G2: Moderate	G3: Severe	G4: Mild	G5: Moderate	G6: Severe	
	(n = 18)	(n = 70)	(n = 178)	(n = 52)	(n = 29)	(n = 24)	***P***-value ^1^
**Daytime sleepiness measurements**							
ESS score	11.09 ± 1.31	9.91 ± 0.66	11.06 ± 0.43	9.65 ± 0.8	10.55 ± 1.02	10.24 ± 1.15	0.618
**Overnight polysomnography**							
AHI, episodes/hour	12.68 ± 3.06	24.41 ± 1.55^a^	55.98 ± 1.01^a,b^	11.93 ± 1.86	21.97 ± 2.39^a^	44.41 ± 2.69^a,b,c^	<0.001*
Supine AHI, episodes/hour	17.15 ± 3.54	33.98 ± 1.80^a^	66.11 ± 1.16^a,b^	18.55 ± 2.13	34.88 ± 2.76^a^	54.55 ± 3.1^a,b,c^	<0.001*
Lateral AHI, episodes/hour	10.26 ± 4.13	14.35 ± 2.10	39.29 ± 1.35^a,b^	3.73 ± 2.48	2.25 ± 3.22^c^	5.17 ± 3.62^c^	<0.001*
DI, episodes/hour	7.07 ± 3.38	15.12 ± 1.95	45.62 ± 1.27^a,b^	7.97 ± 2.33	14.90 ± 3.00	35.91 ± 3.37^a,b^	<0.001*
AI, episodes/hour	30.87 ± 3.66	36.78 ± 1.86	56.65 ± 1.21^a,b^	19.50 ± 2.23	27.91 ± 2.86	46.99 ± 3.23^a,b^	<0.001*
Sleep efficiency, %	68.83 ± 3.61	74.9 ± 1.84	77.62 ± 1.19	77.16 ± 2.2	78.46 ± 2.82	78.48 ± 3.18	0.224
Sleep latency, minute	28.85 ± 7.06	27.53 ± 3.59	22.3 ± 2.33	23.64 ± 4.3	16.58 ± 5.52	24.6 ± 6.22	0.601
REM latency, minute	120.2 ± 17.1	134.32 ± 8.83	141.17 ± 5.7	113.89 ± 10.29	126.42 ± 12.99	147.72 ± 15.25	0.238
S1, %	22.22 ± 3.18	24.01 ± 1.62	36.19 ± 1.05^a,b^	20.33 ± 1.94	23.30 ± 2.49	28.61 ± 2.8	<0.001*
S2, %	62.00 ± 3.16	60.83 ± 1.60	51.52 ± 1.04^a,b^	60.52 ± 1.92	59.14 ± 2.47	57.84 ± 2.78	<0.001*
S34, %	3.18 ± 1.35	2.72 ± 0.69	2.5 ± 0.44	4.01 ± 0.82	4.13 ± 1.06	2.61 ± 1.19	0.560
REM, %	12.60 ± 1.54	12.43 ± 0.78	9.78 ± 0.51	15.14 ± 0.94	13.43 ± 1.20	10.94 ± 1.35	<0.001*
SaO_2_ mean, %	92.92 ± 0.73	93.23 ± 0.37	90.65 ± 0.24^b^	93.91 ± 0.45	94.14 ± 0.57	93.66 ± 0.65^c^	<0.001*
SaO_2_ minimal, %	86.36 ± 1.90	81.22 ± 0.97^a^	76.02 ± 0.63^a,b^	84.56 ± 1.17	81.87 ± 1.49	76.96 ± 1.67^a^	<0.001*
**Blood pressure**							
Evening SBP, mmHg	125.28 ± 3.43	127.46 ± 1.74	129.23 ± 1.13	124.02 ± 2.09	123.05 ± 2.68	126.01 ± 3.02	0.176
Evening DBP, mmHg	76.84 ± 2.49	77.15 ± 1.27	79.29 ± 0.82	76.47 ± 1.52	73.78 ± 1.95	78.76 ± 2.19	0.138
Evening MABP, mmHg	92.99 ± 2.61	93.92 ± 1.33	95.93 ± 0.86	92.32 ± 1.59	90.2 ± 2.04	94.51 ± 2.3	0.119
Morning SBP, mmHg	125.91 ± 3.27	126.83 ± 1.66	132.44 ± 1.08	125.06 ± 1.99	122.72 ± 2.56	126.56 ± 2.88	0.002*
Morning DBP, mmHg	78.37 ± 2.83	79.62 ± 1.44	83.74 ± 0.93^b^	79.06 ± 1.72	77.32 ± 2.21	80.81 ± 2.49	0.033*
Morning MABP, mmHg	94.23 ± 2.79	95.35 ± 1.42	99.97 ± 0.92	94.39 ± 1.70	92.47 ± 2.18	96.07 ± 2.46	0.006*
Evening-to-morning SBP difference, mmHg	0.63 ± 2.67	-0.63 ± 1.36	3.21 ± 0.88	1.04 ± 1.63	-0.33 ± 2.09	0.54 ± 2.36	0.234
Evening-to-morning DBP difference, mmHg	1.53 ± 2.15	2.47 ± 1.09	4.45 ± 0.71	2.59 ± 1.31	3.54 ± 1.68	2.05 ± 1.89	0.526
Evening-to-morning MABP difference, mmHg	1.23 ± 2.11	1.43 ± 1.07	4.04 ± 0.7	2.07 ± 1.28	2.26 ± 1.65	1.55 ± 1.86	0.338

ESS: Epworth Sleepiness Scale; AHI: apnea-hypopnea index; DI: desaturation index; AI: arousal index; REM: rapid eye movement; SaO2: oxygen saturation; SBP: systolic blood pressure; DBP: diastolic blood pressure; MABP: mean arterial blood pressure.

Results are estimated mean ± standard deviation.

*Indicates a significant association, *P* < 0.05.

^1^After adjusting for gender, ratio of waist to hip, BMI, smoking, and cardiovascular comorbidity, associations between factors and groups were assessed by linear regression and *P*-values were estimated using Wald’s test. Post-hoc testes were examined by Boferroni correction.

^a^Indicates significant difference from the mild subgroup within the nonpositional (or positional) group, *p* < 0.05.

^b^Indicates significant difference from the moderate subgroup within the nonpositional (or positional) group, *p* < 0.05.

^c^Indicates significant difference between the corresponding subgroups of nonpositional and positional group, *p* < 0.05.

### Blood pressure

As shown in Table 2, morning BP differed across all groups (*P* = 0.002 for morning SBP, *P* =0.033 for morning DBP, *P* = 0.006 for morning MABP). BP was similar between non-positional and positional group; however, the morning DBP in the group 2 was lower compared with group 3.

### CPAP acceptance and compliance

Table 3 shows CPAP acceptance and compliance. Mild OSA patients showed the lowest CPAP acceptance rate (10%), followed by moderate group (37.37%) and severe group (61.88%, *P* < 0.001). However, the significant difference in CPAP acceptance across OSA severity disappeared when the data was stratified by positional dependency. The duration of follow-up varied across groups ranging from 15.5 to 47 months.

**Table 3:** **Continuous positive airway pressure acceptance and compliance among patients with non-positional and positional obstructive sleep apnea**

Outcome	Mild		Moderate		Severe		***P***-value for comparison among severity levels
	Non-positional	Positional	Non-positional	Positional	Non-positional	Positional	
	(n = 18)	(n = 52)	(n = 70)	(n = 29)	(n = 178)	(n = 24)	
CPAP acceptance by severity	7 (10.00%)		37 (37.37%)		125 (61.88%)		<0.001*
CPAP acceptance in each group	1 (5.56%)	6 (11.54%)	26 (37.14%)	11 (37.93%)	110 (61.80%)	15 (62.50%)	
CPAP compliance^1^							0.896
High	1 (100%)	4 (66.67%)	18 (69.23%)	7 (63.64%)	79 (71.81%)	11 (73.33%)	
Low	0 (0%)	2 (33.33%)	8 (30.77%)	4 (36.36%)	31 (28.19%)	4 (26.67%)	
Follow-up duration, months^1^	47.00	15.50 ± 9.57	20.62 ± 15.58	23.09 ± 19.54	22.27 ± 15.58	17.07 ± 14.68	NA

CPAP: continuous positive airway pressure, NA: not available due to only one case in one group.

Results are number (percentage) for categorical variables and mean ± standard deviation for continuous variables. Chi-square test or Fisher’s exact test was implemented for categorical variables.

^1^Statistics were derived from CPAP acceptors.

## Discussion

This study found that positional mild OSA had less CV co-morbidities and non-positional moderate group had lower morning BP compared with severe OSA. In addition, OSA showed varying associations with sleep parameters depending upon severity and positional dependence. Patients with mild OSA had low CPAP acceptance but similar CPAP compliance compared with patients who had moderate or severe OSA. CPAP acceptance was not dependent on posture dependency of OSA.

The prevalence of positional OSA in our study among patients with mild OSA was 74.3% (52 of 70 mild OSA patients). This percentage is similar to that reported in a previous study using the same definitions for positional-dependency [22]. Results of overnight PSG studies showed that patients with mild OSA in the positional group had lower AI and better sleep efficiency than the patients with mild OSA in the non-positional group, which also supports the results of a previous study [8]. AI was within normal limits for our positional mild OSA group, but was higher than normal in the non-positional mild OSA group. Sleep efficiency was lower than normal in both groups. While associations between OSA and hypertension have been generally accepted, studies have also shown that sleep-disordered breathing, short sleep, and poor sleep have been associated with hypertension in the general population [23–25]. Therefore, patients experiencing poor sleep quality should receive optimal treatment (including surgery or CPAP), regardless of OSA severity or positional dependency in order to reduce the risk of developing hypertension.

The prevalence of hypertension in both mild OSA groups (61.1% of non-positional mild OSA and 34.6% of positional mild OSA patients) was similar to that reported in a previous study [22] and was higher in both groups than the prevalence in general population [26]. Much evidence supports elevated BP as a predictor of CV disease. Both increased SBP and increased DBP have been reported to be significant predictors of stroke and coronary artery disease [27]. On average, SBP and DBP readings in the morning are 10%-20% lower than BP readings in the evening [28]. A recent study showed that when this morning dip is replaced by a morning surge in patients with hypertension, the risk of developing a major CV event rises significantly [29]. Ting et al. reported that elevated morning SBP was associated with significantly greater respiratory disturbances, blood glucose, and metabolic syndrome score [30]. Many patients with OSA exhibit loss of the overnight dip in BP and exhibit morning BP elevations, possibly due to sympathetic nervous system over activity [31, 32]. In this study, patients in both the positional and non-positional groups showed a loss of evening to morning dip in BP, which supports the aforementioned findings. Our finding suggests that patients with positional mild OSA may have the same level of risk of developing CV disease as patients with non-positional mild OSA. These results suggest that there may be benefit in treating patients with mild OSA regardless of positional dependency. Well-designed randomized controlled trials are needed to directly further address these issues.

Several studies have documented that poor sleep quality is closely associated with hypertension [23–25]. There was no evidence that improving sleep lowers blood pressure, until recently. Huang et al. [33] found that the blood pressure of poor sleepers was significantly reduced compared to pretreatment values following zolpidem (a non-benzodiazepine, non-hypotensive mild sedative with a short elimination half-life of 2.5 hrs) treatment (P < 0.05) and more poor sleepers treated with zolpidem were converted from nondipping hypertension to dipping hypertension. Zolpidem treatment did not affect the blood pressure of good sleepers. Additional randomized controlled studies as necessary to further explore the potential that improving sleep, possibly through the use of CPAP, can reduce blood pressure.

Positional therapy is often relied upon as the primary treatment modality for positional mild OSA. A variety of strategies have been proposed for keeping patients in a non-supine position, including the use of tennis balls, vibrating positional alarms, and wearable devices [34, 35]. However, while positional therapy can moderately reduce AHI in some patients, long-term compliance with positional therapy is poor as outlined in recent reviews [10, 36]. In addition, since CPAP clearly provides superior improvement over positional therapy, positional therapy is not recommended for first-line treatment of OSA [10].

The efficacy of CPAP in reducing apnea symptoms and the risk of CV disorders has been clearly demonstrated, making it the treatment of choice for patients with OSA; however, patients are often reluctant to accept CPAP treatment due to perceived inconvenience and discomfort [37]. Unsurprisingly, few patients in our study were willing to start CPAP treatment, especially in the mild OSA patients (10%). However, the overall CPAP acceptance of all the patients was 45.6%, and for patients with mild OSA was 10%. The low acceptance rate may, in part reflect the small sample size and the fact that in Taiwan, the cost of CPAP is not covered by national or commercial insurance and is paid out-of-pocket by the patient and is 2–3 times higher than that in other countries [38]. Acceptance rates from other studies range from 40-90% [38–45]. In the studies in which the acceptance rate is higher (about 70%), often CPAP is free to subject participating in these trials [42–45]. Other reasons that may have affected the acceptance rate are that the retrospective study design is closer to the real-world setting and that the strong national health insurance and healthcare in Taiwan is so convenient and inexpensive that the Taiwanese pay little attention on health maintenance. Overall CPAP compliance of our patients was 71.4% (120 out of 168 patients) and is comparable with that found in studies in Europe and US which were about 75% and 46%, respectively [42, 43, 46–49]. Reasons for the wide range of compliance across countries include different definition of compliance and different sources of CPAP. Moreover, free CPAP in prospective studies results in higher compliance, while out-of-pocket CPAP in retrospective studies results in lower compliance. Nonetheless, the majority of our patients who accepted CPAP treatment showed a high level of compliance. Thus, educational support and physician involvement to increase patient awareness of the increased CV risks, even with mild OSA, and the potential benefits of CPAP treatment for CV health might improve patient acceptance and compliance [41].

There is growing evidence supporting the use of CPAP in sleepy patients with mild OSA [50], however, the findings are mixed and CPAP treatment of mild OSA is recommended as only a treatment option [51]. Given the findings that there is an association of mild OSA with hypertension and mild OSA tends to worse over time, we suggest that CPAP treatment may benefit these patients. However, further randomized controlled studies are required to better understand the potential benefit of CPAP in treating mild OSA and hypertension.

This study is limited by the retrospective design and the small number of patients included. Larger controlled studies are warranted to further study the effects of positional dependency on BP, and CPAP efficacy and usage patterns in patients with OSA. Besides, we used a thermistor (not nasal pressure transducer to detect airflow and not an esophageal pressure sensor to detect respiratory effort related arousal). These may result in an under-estimation of the severity of OSA and consequently misclassification of some patients in the mild and moderate OSA groups.

## Conclusion

This study found that positional mild OSA had less cardiovascular co-morbidities and non-positional moderate OSA had lower morning blood pressure compared with severe group. CPAP acceptance by patients with mild OSA was low, but CPAP compliance was similar to that of patients with moderate or severe OSA. CPAP acceptance rate was independent of posture dependency of OSA. Since there is increasing evidences of greater cardiovascular risk for untreated mild OSA, optimal treatment (including CPAP or surgery) for mild OSA regardless of posture dependency is critical. Strategies to improving CPAP acceptance among mild OSA patients will be the issue in the future.

## Abbreviations

OSA: Obstructive sleep apnea
CPAP: Continuous positive airway pressure
CV: Cardiovascular
PSG: Polysomnography
AHI: Apnea-hypopnea index
BP: Blood pressure
ESS: Epworth sleepiness scale
EEG: Electroencephalography
SBP: Systolic BP
DBP: Diastolic BP
MABP: Mean arterial blood pressure
BMI: body mass index
CAD: Coronary artery disease
CVA: Cerebrovascular accident
DI: Desaturation index
AI: Arousal index
REM: Rapid eye movement
SaO2: Oxygen saturation.

## References

[1] Thomas JJ, Ren J (2012). Obstructive sleep apnea and cardiovascular complications: perception versus knowledge. Clin Exp Pharmacol Physiol.

[2] Peppard PE, Young T, Palta M, Skatrud J (2000). Prospective study of the association between sleep-disordered breathing and hypertension. N Engl J Med.

[3] Drager LF, Bortolotto LA, Lorenzi MC, Figueiredo AC, Krieger EM, Loranzi-Filho G (2005). Early signs of atherosclerosis in obstructive sleep apnea. Am J Respir Crit Care Med.

[4] Shahar E, Whitney CW, Redlines S, Lee ET, Newman AB, Nieto FJ, O’connor GT, Boland LL, Schwartz JE, Samet JM (2001). Sleep-disordered breathing and cardiovascular disease: cross-sectional results of the Sleep Heart Health Study. Am J Respir Crit Care Med.

[5] Jaimchariyatam N, Rodriguez CL, Budur K (2010). Does CPAP treatment in mild obstructive sleep apnea affect blood pressure?. Sleep Med.

[6] Barbé F, Durán-Cantolla J, Sánchez-de-la-Torre M, Martínez-Alonso M, Carmona C, Barceló A, Chiner E, Masa JF, Gonzalez M, Marín JM, Garcia-Rio F, Diaz de Atauri J, Terán J, Mayos M, de la Peña M, Monasterio C, del Campo F, Montserrat JM, Spanish Sleep And Breathing Network (2012). Effect of continuous positive airway pressure on the incidence of hypertension and cardiovascular events in nonsleepy patients with obstructive sleep apnea: a randomized controlled trial. JAMA.

[7] Sahlman J, Pukkila M, Seppa J, Tuomilehto H (2007). Evolution of mild obstructive sleep apnea after different treatments. Laryngoscope.

[8] Oksenberg A, Silverberg DS, Arons E, Radwan H (1997). Positional vs nonpositional obstructive sleep apnea patients: anthropomorphic, nocturnal polysomnographic, and multiple sleep latency test data. Chest.

[9] Isono S, Tanaka A, Nishino T (2002). Lateral position decreases collapsibility of the passive pharynx in patients with obstructive sleep apnea. Anesthesiology.

[10] Randerath WJ, Verbraecken J, Andreas S, Bettega G, Boudewyns A, Hamans E, Jalbert F, Paoli JR, Sanner B, Smith I, Stuck BA, Lacassagne L, Marklund M, Maurer JT, Pepin JL, Valipour A, Verse T, Fietze I, European Respiratory Society task force on non-CPAP therapies in sleep apnoea (2011). Non-CPAP therapies in obstructive sleep apnoea. Eur Respir J.

[11] Giles TL, Lasserson TJ, Smith BH, White J, Wright J, Cates CJ (2006). Continuous positive airways pressure for obstructive sleep apnoea in adults. Cochrane Database Syst Rev.

[12] Budhiraja R, Budhiraja P, Quan SF (2010). Sleep-disordered breathing and cardiovascular disorders. Respir Care.

[13] Buchner NJ, Quack I, Stegbauer J, Woznowski M, Kaufmann A, Rump LC (2012). Treatment of obstructive sleep apnea reduces arterial stiffness. Sleep Breath.

[14] Drager LF, Pedrosa RP, Diniz PM, Diegues-Silva L, Marcondes B, Couto RB, Giorgi DM, Krieger EM, Lorenzi-Filho G (2011). The effects of continuous positive airway pressure on prehypertension and masked hypertension in men with severe obstructive sleep apnea. Hypertension.

[15] Mador MJ, Kufel TJ, Magalang UJ, Rajesh SK, Watwe V, Grant BJ (2005). Prevalence of positional sleep apnea in patients undergoing polysomnography. Chest.

[16] Chen NH, Johns MW, Li HY, Chu CC, Liang SC, Shu YH, Chuang ML, Wang PC (2002). Validation of a Chinese version of the Epworth sleepiness scale. Qual Life Res.

[17] Rechtschaffen A, Kales A (1968). A manual of standardized terminology, techniques and scoring system for sleep stages of human subjects.

[18] Boselli M, Parrino L, Smerieri A, Terzano MG (1998). Effect of age on EEG arousals in normal sleep. Sleep.

[19] Anstead M, Phillips B, Buch K (1998). Tolerance and intolerance to continuous positive airway pressure. Curr Opin Pulm Med.

[20] Grunstein RR (1995). Sleep-related breathing disorders. 5. Nasal continuous positive airway pressure treatment for obstructive sleep apnoea. Thorax.

[21] Gay P, Weaver T, Loube D, Iber C, Positive Airway Pressure Task Force; Standards of Practice Committee; American Academy of Sleep Medicine (2006). Evaluation of positive airway pressure treatment for sleep related breathing disorders in adults. Sleep.

[22] Mo JH, Lee CH, Rhee CS, Yoon IY, Kim JW (2011). Positional dependency in Asian patients with obstructive sleep apnea and its implication for hypertension. Arch Otolaryngol Head Neck Surg.

[23] Bansil P, Kuklina EV, Merritt RK, Yoon PW (2011). Associations between sleep disorders, sleep duration, quality of sleep, and hypertension: results from the National Health and Nutrition Examination Survey, 2005 to 2008. J Clin Hypertens (Greenwich).

[24] Gangwisch JE, Heymsfield SB, Boden-Albala B, Buijs RM, Kreier F, Pickering TG, Rundle AG, Zammit GK, Malaspina D (2006). Short sleep duration as a risk factor for hypertension: analyses of the first National Health and Nutrition Examination Survey. Hypertension.

[25] Pepin JL, Borel AL, Tamisier R, Baguet JP, Levy P, Dauvilliers Y (2014). Hypertension and sleep: Overview of a tight relationship. Sleep Med Rev.

[26] Hajjar I, Kotchen JM, Kotchen TA (2006). Hypertension: trends in prevalence, incidence, and control. Annu Rev Public Health.

[27] Somers VK, White DP, Amin R, Abraham WT, Costa F, Culebras A, Daniels S, Floras JS, Hunt CE, Olson LJ, Pickering TG, Russell R, Woo M, Young T, American Heart Association Council for High Blood Pressure Research Professional Education Committee, Council on Clinical Cardiology; American Heart Association Stroke Council; American Heart Association Council on Cardiovascular Nursing; American College of Cardiology Foundation (2008). Sleep apnea and cardiovascular disease: an American Heart Association/american College Of Cardiology Foundation Scientific Statement from the American Heart Association Council for High Blood Pressure Research Professional Education Committee, Council on Clinical Cardiology, Stroke Council, and Council On Cardiovascular Nursing. In collaboration with the National Heart, Lung, and Blood Institute National Center on Sleep Disorders Research (National Institutes of Health). Circulation.

[28] Kanbay M, Turgut F, Uyar ME, Akcay A, Covic A (2008). Causes and mechanisms of nondipping hypertension. Clin Exp Hypertens.

[29] Verdecchia P, Angeli F, Mazzotta G, Garofoli M, Ramundo E, Gentile G, Ambrosio G, Reboldi G (2012). Day-night dip and early-morning surge in blood pressure in hypertension: prognostic implications. Hypertension.

[30] Ting H, Lo HS, Chang SY, Chung AH, Kuan PC, Yuan SC, Huang CN, Lee SD (2009). Post- to pre-overnight sleep systolic blood pressures are associated with sleep respiratory disturbance, pro-inflammatory state and metabolic situation in patients with sleep-disordered breathing. Sleep Med.

[31] Dopp JM, Reichmuth KJ, Morgan BJ (2007). Obstructive sleep apnea and hypertension: mechanisms, evaluation, and management. Curr Hypertens Rep.

[32] Hoffstein V, Mateika J (1992). Evening-to-morning blood pressure variations in snoring patients with and without obstructive sleep apnea. Chest.

[33] Huang Y, Mai W, Cai X, Hu Y, Song Y, Qiu R, Wu Y, Kuang J (2012). The effect of zolpidem on sleep quality, stress status, and nondipping hypertension. Sleep Med.

[34] van Maanen JP, Meester KA, Dun LN, Koutsourelakis I, Witte BI, Laman DM, Hilgevoord AA, de Vries N (2013). The sleep position trainer: a new treatment for positional obstructive sleep apnoea. Sleep Breath.

[35] Bignold JJ, Deans-Costi G, Goldsworthy MR, Robertson CA, McEvoy D, Catcheside PG, Mercer JD (2009). Poor long-term patient compliance with the tennis ball technique for treating positional obstructive sleep apnea. J Clin Sleep Med.

[36] Smith I, Nadig V, Lasserson TJ (2009). Educational, supportive and behavioural interventions to improve usage of continuous positive airway pressure machines for adults with obstructive sleep apnoea. Cochrane Database Syst Rev.

[37] Mannarino MR, Di Filippo F, Pirro M (2012). Obstructive sleep apnea syndrome. Eur J Intern Med.

[38] Simon-Tuval T, Reuveni H, Greenberg-Dotan S, Oksenberg A, Tal A, Tarasiuk A (2009). Low socioeconomic status is a risk factor for CPAP acceptance among adult OSAS patients requiring treatment. Sleep.

[39] Engleman HM, Martin SE, Deary IJ, Douglas NJ (1997). Effect of CPAP therapy on daytime function in patients with mild sleep apnoea/hypopnoea syndrome. Thorax.

[40] Engleman HM, Kingshott RN, Wraith PK, Mackay TW, Deary IJ, Douglas NJ (1999). Randomized placebo-controlled crossover trial of continuous positive airway pressure for mild sleep Apnea/Hypopnea syndrome. Am J Respir Crit Care Med.

[41] Tarasiuk A, Reznor G, Greenberg-Dotan S, Reuveni H (2012). Financial incentive increases CPAP acceptance in patients from low socioeconomic background. PLoS One.

[42] Abdelghani A, Slama S, Hayouni A, Harrabi I, Mezghanni S, Garrouche A, Klabi N, Benzarti M, Jerray M (2009). Acceptance and long-term compliance to continuous positive airway pressure in obstructive sleep apnea. A prospective study on 72 patients treated between 2004 and 2007 [Article in French]. Rev Pneumol Clin.

[43] Richard W, Venker J, den Herder C, Kox D, van den Berg B, Laman M, van Tinteren H, de Vries N (2007). Acceptance and long-term compliance of nCPAP in obstructive sleep apnea. Eur Arch Otorhinolaryngol.

[44] Gordon P, Sanders MH (2005). Sleep.7: positive airway pressure therapy for obstructive sleep apnoea/hypopnoea syndrome. Thorax.

[45] Reeves-Hoche MK, Meck R, Zwillich CW (1994). Nasal CPAP: an objective evaluation of patient compliance. Am J Respir Crit Care Med.

[46] Collard P, Pieters T, Aubert G, Delguste P, Rodenstein DO (1997). Compliance with nasal CPAP in obstructive sleep apnea patients. Sleep Med Rev.

[47] Kakkar RK, Berry RB (2007). Positive airway pressure treatment for obstructive sleep apnea. Chest.

[48] Boyacı H, Gacar K, Barış SA, Başyiğit I, Yıldız F (2013). Positive airway pressure device compliance of the patients with obstructive sleep apnea syndrome. Adv Clin Exp Med.

[49] Pieters T, Collard P, Aubert G, Dury M, Delguste P, Rodenstein DO (1996). Acceptance and long-term compliance with nCPAP in patients with obstructive sleep apnoea syndrome. Eur Respir J.

[50] Peker Y (2012). Growing research evidence for continuous positive airway pressure treatment for sleepy patients with milder obstructive sleep apnea. Am J Respir Crit Care Med.

[51] Kushida CA, Littner MR, Hirshkowitz M, Morgenthaler TI, Alessi CA, Bailey D, Boehlecke B, Brown TM, Coleman J, Friedman L, Kapen S, Kapur VK, Kramer M, Lee-Chiong T, Owens J, Pancer JP, Swick TJ, Wise MS, American Academy of Sleep Medicine (2006). Practice parameters for the use of continuous and bilevel positive airway pressure devices to treat adult patients with sleep-related breathing disorders. Sleep.

[CR52] The pre-publication history for this paper can be accessed here:http://www.biomedcentral.com/1471-2466/14/153/prepub

